# P-58 - TUBERCULOSIS CASES REPORTED TO THE PUBLIC HEALTH AGENCY IN NORTHERN IRELAND: A RETROSPECTIVE ANALYSIS OF NOTIFICATION SOURCES AND TIMELINESS OVER 1-YEAR PERIOD

**DOI:** 10.1093/pubmed/fdag046.088

**Published:** 2026-07-09

**Authors:** Dr Corey Magee, Dr Jillian Johnston

**Affiliations:** Public Health Agency; Public Health Agency

## Abstract

**Background:**

Tuberculosis (TB) is a leading global cause of preventable infectious deaths. Timely notification of TB cases is essential to facilitate prompt public health action and prevent onward transmission. This study reviewed TB notifications reported to the Public Health Agency (PHA) in Northern Ireland (NI) over a 1-year period to describe notification sources and timeliness.

**Methods:**

We conducted a retrospective quantitative analysis of all TB cases reported to the PHA between 1 November 2024 and 31 October 2025. In NI, TB notification forms (TBS1) serve a dual purpose for both notification and collection of surveillance information. Data were extracted from the HP Zone case management system. Timeliness of initial notification was calculated as the interval from diagnosis to initial notification, and TBS1 timeliness as the interval from notification to TBS1 receipt. Timeliness of contact tracing initiation was measured as the interval between issuing a standard letter to specialist trust TB nurses and the date of initial notification and TBS1 submission, respectively.

**Results:**

Eighty-nine TB cases were included. Laboratory reports were the most common initial notification source (55%), while 35% of cases were first notified through clinician-submitted TBS1 forms. Overall, 50.5% were notified within three days of diagnosis, and 54% of TBS1 forms were submitted within three days. The median interval between initial notification and a formal request to initiate contact tracing was six days.

**Conclusions:**

This study provides insight into the timeliness of TB notifications in NI. Most cases were notified only when confirmed, despite legislation requiring notification at the suspected stage. Laboratory reports, though not mandated, were the most common initial notification source. Delays in notification and TBS1 submission highlight a need to balance timely notification against the need for more complete case data to support a timely public health response.

